# 1,2,3-Trifluoro­benzene

**DOI:** 10.1107/S1600536809038975

**Published:** 2009-10-07

**Authors:** Michael T. Kirchner, Dieter Bläser, Roland Boese, Tejender S. Thakur, Gautam R. Desiraju

**Affiliations:** a Institut für Anorganische Chemie der Universität, 45117 Essen, Germany; b Indian Institute of Science, Bangalore 560 012, India

## Abstract

In the title compound, C_6_H_3_F_3_, weak electrostatic and dispersive forces between C(δ+)—F(δ−) and H(δ+)—C(δ−) groups are at the borderline of the hydrogen-bond phenomenon and are poorly directional and further deformed in the presence of π–π stacking inter­actions. The mol­ecule lies on a twofold rotation axis. In the crystal structure, one-dimensional tapes are formed *via* two anti­dromic C—H⋯F hydrogen bonds. These tapes are, in turn, connected into corrugated two-dimensional sheets by bifurcated C—H⋯F hydrogen bonds. Packing in the third dimension is furnished by π–π stacking inter­actions with a centroid–centroid distance of 3.6362 (14) Å.

## Related literature

For C—H⋯F inter­actions, see: Althoff *et al.* (2006 ▶); Bats *et al.* (2000 ▶); Choudhury *et al.* (2004 ▶); D’Oria & Novoa (2008 ▶); Dunitz & Taylor (1997 ▶); Howard *et al.* (1996 ▶); Müller *et al.* (2007 ▶); O’Hagan (2008 ▶); Reichenbacher *et al.* (2005 ▶); Weiss *et al.* (1997 ▶). For related crystal structures of several polyfluorinated benzenes, see: Thalladi *et al.* (1998 ▶). For crystallization techniques, see: Boese & Nussbaumer (1994 ▶).
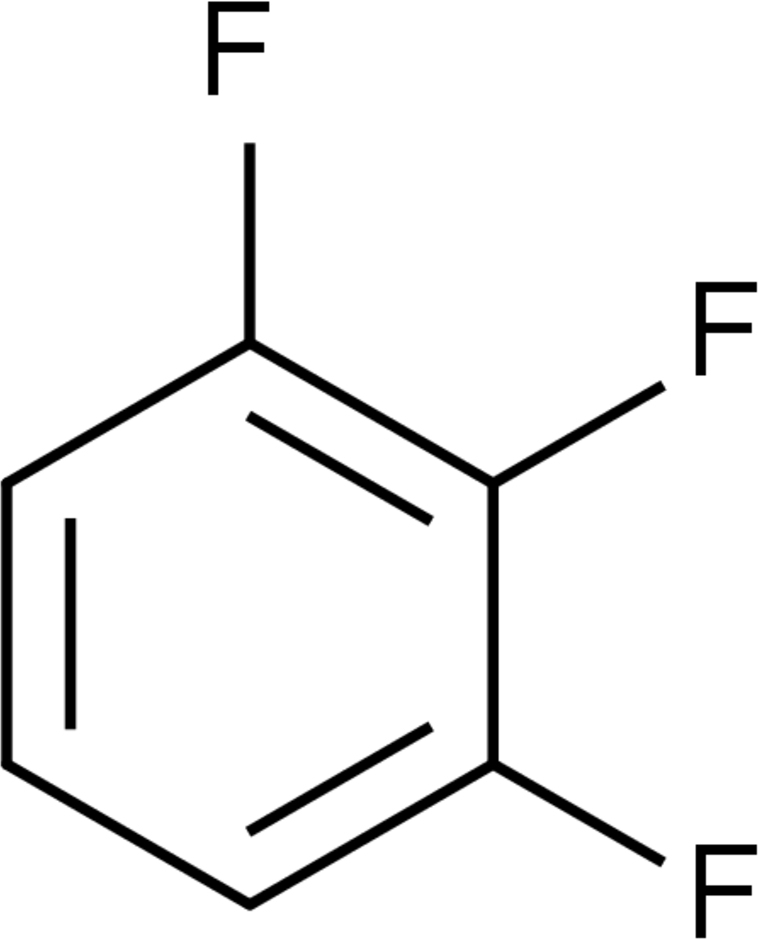


## Experimental

### 

#### Crystal data

C_6_H_3_F_3_
*M* *_r_* = 132.08
Monoclinic, 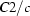

*a* = 7.4238 (19) Å
*b* = 11.590 (3) Å
*c* = 7.0473 (17) Å
β = 112.783 (4)°
*V* = 559.1 (2) Å^3^
*Z* = 4
Mo *K*α radiation
μ = 0.16 mm^−1^
*T* = 233 K
0.30 × 0.30 × 0.30 mm

#### Data collection

Siemens SMART three-axis goniometer with an APEXII area-detector system diffractometer
Absorption correction: multi-scan (*SADABS*; Bruker; 2004 ▶) *T*_min_ = 0.820, *T*_max_ = 0.953
1074 measured reflections
634 independent reflections
413 reflections with *I* > 2σ(*I*)
*R*_int_ = 0.013

#### Refinement

*R*[*F* ^2^ > 2σ(*F* ^2^)] = 0.061
*wR*(*F* ^2^) = 0.226
*S* = 1.04
634 reflections
44 parameters
H-atom parameters constrained
Δρ_max_ = 0.20 e Å^−3^
Δρ_min_ = −0.18 e Å^−3^

### 

Data collection: *APEX2* (Bruker, 2008 ▶); cell refinement: *SAINT* (Bruker, 2008 ▶); data reduction: *SAINT*; program(s) used to solve structure: *SHELXTL* (Sheldrick, 2008); program(s) used to refine structure: *SHELXTL*; molecular graphics: *Mercury* (Macrae *et al.*, 2008 ▶) and *GIMP2* (The GIMP team, 2008 ▶); software used to prepare material for publication: *publCIF* (Westrip, 2009 ▶).

## Supplementary Material

Crystal structure: contains datablocks I, global. DOI: 10.1107/S1600536809038975/lh2880sup1.cif

Structure factors: contains datablocks I. DOI: 10.1107/S1600536809038975/lh2880Isup2.hkl

Additional supplementary materials:  crystallographic information; 3D view; checkCIF report

## Figures and Tables

**Table 1:** Hydrogen-bond geometry (Å, °)

*D*—H⋯*A*	*D*—H	H⋯*A*	*D*⋯*A*	*D*—H⋯*A*
C3—H3⋯F2^i^	1.10	2.77	3.560 (3)	129
C3—H3⋯F1^ii^	1.10	2.59	3.528 (4)	144
C4—H4⋯F2^iii^	1.00	2.60	3.440 (4)	142

Symmetry codes: (i) 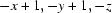; (ii) 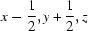; (iii) 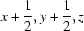.

**Fig. 1.**
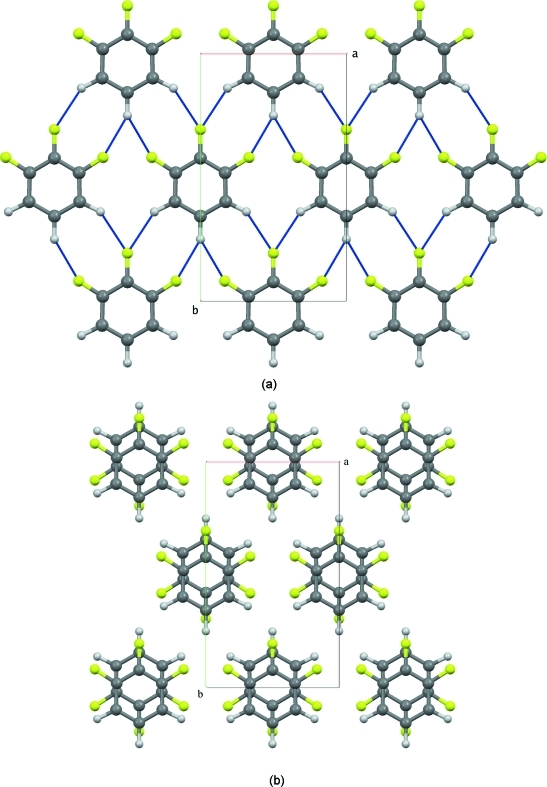
Part of the crystal structure of 1 (a) 2D network of C–H···F–C interactions viewed along the c-axis (b) π–π stacking of molecules viewed along the c-axis.

**Fig. 2.**
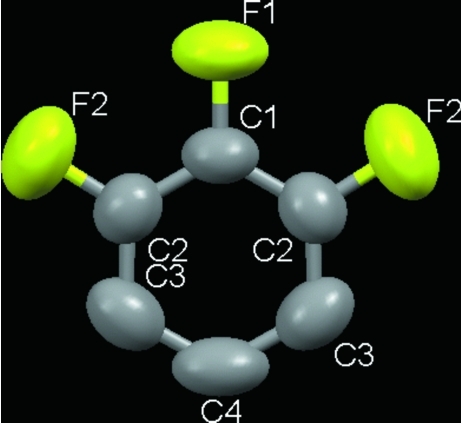
The molecular structure of (1) with displacement ellipsoids drawn at the 50% probability level. The identically labelled atoms are related to each other by the symmetry operator (2-x, y, -z+1/2).

## supplementary crystallographic information

### Comment

Despite the high electronegativity difference between carbon and fluorine, the C–F bond acts as a poor hydrogen bond acceptor due to the hardness of the F-atom (Dunitz & Taylor, 1997; O'Hagan, 2008). The resultant weak C–H···F–C interactions (Howard *et al.*, 1996; Reichenbacher *et al.*, 2005) arise mainly due to electrostatic and dispersive forces between the C(δ+)–F(δ-) and the H(δ+)–C(δ-) fragments. These interactions, at the borderline of the hydrogen bond phenomenon, are also poorly directional and are deformed by other dominant interactions (Weiss, *et al.*, 1997; D'Oria & Novoa, 2008; Müller *et al.*, 2007). In the absence of other interactions these weak interactions can play a role in the overall crystal packing of the molecule (Bats *et al.* 2000; Choudhury *et al.* 2004; Althoff *et al.* 2006). In activated systems such as polyfluorobenzenes, C–H···F–C interactions may be of significance, and in connection there are some reports of the crystal structures of several polyfluorinated benzene compunds (Thalladi *et al.*, 1998). As a continuation of this work, we report here the crystal structure 1,2,3-trifluorobenzene (1). The comparison crystal structures of 1,2- and 1,4-difluorobenzene and 1,3,5-trifluorobenzene have been reported in this earlier work.

### Experimental

The crystals were prepared from commerical samples by zone melting in a quartz capillary at 235 K (1) according to the procedure outlined by (Boese & Nussbaumer, 1994).

### Refinement

Treatment of hydrogen atoms: Riding model with the 1.2 fold isotropic displacement parameters of the equivalent *U^ij^* of the corresponding carbon atom.

### Crystal data

**Table:**

C_6_H_3_F_3_	*F*(000) = 264
*M**_r_* = 132.08	*D*_x_ = 1.569 Mg m^−^^3^
Monoclinic, *C*2/*c*	Mo *K*α radiation, λ = 0.71073 Å
Hall symbol: -C 2yc	Cell parameters from 376 reflections
*a* = 7.4238 (19) Å	θ = 3.8–22.7°
*b* = 11.590 (3) Å	µ = 0.16 mm^−^^1^
*c* = 7.0473 (17) Å	*T* = 233 K
β = 112.783 (4)°	Cylindric, colourless
*V* = 559.1 (2) Å^3^	0.30 × 0.30 × 0.30 mm
*Z* = 4	

### Data collection

**Table:**

Siemens SMART three-axis goniometer with an APEXII area-detector system diffractometer	634 independent reflections
Radiation source: fine-focus sealed tube	413 reflections with *I* > 2σ(*I*)
graphite	*R*_int_ = 0.013
Detector resolution: 512 pixels mm^-1^	θ_max_ = 28.2°, θ_min_ = 3.5°
in ω at 0.3° scan width one run with 740 frames, phi = 0°, chi = 0°	*h* = −9→9
Absorption correction: multi-scan (*SADABS*; Bruker; 2004)	*k* = −14→10
*T*_min_ = 0.820, *T*_max_ = 0.953	*l* = −9→4
1074 measured reflections	

### Refinement

**Table:**

Refinement on *F*^2^	Primary atom site location: structure-invariant direct methods
Least-squares matrix: full	Secondary atom site location: difference Fourier map
*R*[*F*^2^ > 2σ(*F*^2^)] = 0.061	Hydrogen site location: inferred from neighbouring sites
*wR*(*F*^2^) = 0.226	H-atom parameters constrained
*S* = 1.04	*w* = 1/[σ^2^(*F*_o_^2^) + (0.1501*P*)^2^ + 0.039*P*], where *P* = (*F*_o_^2^ + 2*F*_c_^2^)/3
634 reflections	(Δ/σ)_max_ = 0.017
44 parameters	Δρ_max_ = 0.20 e Å^−^^3^
0 restraints	Δρ_min_ = −0.18 e Å^−^^3^

### Special details

**Table:**

Geometry. All e.s.d.'s (except the e.s.d. in the dihedral angle between two l.s. planes) are estimated using the full covariance matrix. The cell e.s.d.'s are taken into account individually in the estimation of e.s.d.'s in distances, angles and torsion angles; correlations between e.s.d.'s in cell parameters are only used when they are defined by crystal symmetry. An approximate (isotropic) treatment of cell e.s.d.'s is used for estimating e.s.d.'s involving l.s. planes.
Refinement. Refinement of *F*^2^ against ALL reflections. The weighted *R*-factor *wR* and goodness of fit *S* are based on *F*^2^, conventional *R*-factors *R* are based on *F*, with *F* set to zero for negative *F*^2^. The threshold expression of *F*^2^ > σ(*F*^2^) is used only for calculating *R*-factors(gt) *etc*. and is not relevant to the choice of reflections for refinement. *R*-factors based on *F*^2^ are statistically about twice as large as those based on *F*, and *R*- factors based on ALL data will be even larger.

### Fractional atomic coordinates and isotropic or equivalent isotropic displacement parameters (Å^2^)

**Table:**

	*x*	*y*	*z*	*U*_iso_*/*U*_eq_	
F1	1.0000	0.30558 (17)	0.2500	0.1156 (10)	
F2	0.6666 (2)	0.4183 (2)	0.1576 (3)	0.1354 (10)	
C1	1.0000	0.4213 (3)	0.2500	0.0769 (9)	
C2	0.8308 (3)	0.4803 (2)	0.2036 (3)	0.0824 (8)	
C3	0.8265 (4)	0.5973 (3)	0.2023 (3)	0.0942 (9)	
H3	0.6833	0.6388	0.1585	0.113*	
C4	1.0000	0.6558 (3)	0.2500	0.1006 (13)	
H4	1.0000	0.7422	0.2500	0.121*	

### Atomic displacement parameters (Å^2^)

**Table:**

	*U*^11^	*U*^22^	*U*^33^	*U*^12^	*U*^13^	*U*^23^
F1	0.161 (2)	0.0623 (13)	0.1249 (16)	0.000	0.0563 (14)	0.000
F2	0.0959 (12)	0.157 (2)	0.1484 (16)	−0.0341 (10)	0.0415 (10)	0.0067 (12)
C1	0.1030 (19)	0.0573 (16)	0.0725 (15)	0.000	0.0364 (13)	0.000
C2	0.0830 (14)	0.0890 (17)	0.0770 (13)	−0.0101 (9)	0.0327 (10)	0.0013 (9)
C3	0.1073 (17)	0.0935 (18)	0.0858 (15)	0.0277 (12)	0.0419 (12)	0.0094 (10)
C4	0.163 (4)	0.0605 (17)	0.0848 (19)	0.000	0.056 (2)	0.000

### Geometric parameters (Å, °)

**Table:**

F1—C1	1.341 (4)	C3—C4	1.377 (3)
F2—C2	1.342 (3)	C3—H3	1.0973
C1—C2	1.354 (3)	C4—H4	1.0018
C2—C3	1.357 (4)		
			
F1—C1—C2	120.30 (15)	C2—C3—H3	117.3
C2^i^—C1—C2	119.4 (3)	C4—C3—H3	124.4
F2—C2—C3	121.1 (2)	C3—C4—C3^i^	121.0 (3)
F2—C2—C1	117.3 (3)	C3—C4—H4	119.5
C3—C2—C1	121.5 (2)	C3^i^—C4—H4	119.5
C2—C3—C4	118.3 (2)		

Symmetry codes: (i) −*x*+2, *y*, −*z*+1/2.

### Hydrogen-bond geometry (Å, °)

**Table:**

*D*—H···*A*	*D*—H	H···*A*	*D*···*A*	*D*—H···*A*
C3—H3···F2^ii^	1.10	2.77	3.560 (3)	129
C3—H3···F1^iii^	1.10	2.59	3.528 (4)	144
C4—H4···F2^iv^	1.00	2.60	3.440 (4)	142

Symmetry codes: (ii) −*x*+1, −*y*+1, −*z*; (iii) *x*−1/2, *y*+1/2, *z*; (iv) *x*+1/2, *y*+1/2, *z*.
